# Development of gelatin hydrogel nonwoven fabrics (Genocel®) as a novel skin substitute in murine skin defects

**DOI:** 10.1016/j.reth.2022.06.002

**Published:** 2022-06-21

**Authors:** Yuanjiaozi Li, Eiichi Sawaragi, Michiharu Sakamoto, Takashi Nakano, Hiroki Yamanaka, Itaru Tsuge, Kumiko Matsuno, Yasuhiko Tabata, Naoki Morimoto

**Affiliations:** a Department of Plastic and Reconstructive Surgery, Graduate School of Medicine, Kyoto University, Sakyo-ku, Kyoto, Japan; b Laboratory of Biomaterials, Institute for Frontier Life and Medical Sciences, Kyoto University, Sakyo-ku, Kyoto, Japan; c Research and Development Center, The Japan Wool Textile Co., Ltd., Kakogawa, Hyogo, Japan

**Keywords:** Skin substitute, Wound healing, Gelatin hydrogel, Skin defects

## Abstract

**Introduction:**

Genocel is an emerging material, used in cell culture, with high mechanical strength and good cytocompatibility. Based on these characteristics, Genocel is considered a promising skin substitute for wound healing. In this study, we explored the possibility of using Genocel as a skin substitute for murine skin defects and compared it with a conventional skin substitute.

**Methods:**

Sheets of Genocel and Pelnac were applied to skin defects created on the backs of mice. On days 7, 14, and 21, the remaining wound area was evaluated and specimens were harvested for HE, Azan, anti-CD31, CD68, and CD163 staining to assess neoepithelialization, granulation tissue, capillary formation, and macrophage infiltration.

**Results:**

No significant differences in the wound area or neoepithelium length were observed between groups. The number of newly formed capillaries in the Genocel group was significantly higher than that in the Pelnac group on day 7 (p < 0.05). In contrast, granulation tissue formation in the Pelnac group was greater than that in the Genocel group on day 14 (p < 0.05). Regarding macrophage infiltration, the pan-macrophage number, M2 macrophage number, and M2 ratio in the Pelnac group were higher than those in the Genocel group on day 14 (p < 0.05). In other aspects, the two materials displayed comparable behavior.

**Conclusions:**

Genocel can be used as a skin substitute equivalent to the conventional one. In addition, Genocel accelerated capillary formation, which is more appropriate than conventional treatments for chronic skin ulcers, such as diabetic ulcers.

## Highlights

• Gelatin hydrogel nonwoven fabrics, Genocel was used for the first time as a skin substitute for murine skin defects.
• Genocel displayed comparable behavior with Pelnac and accelerated capillary formation in the early phase.
• However, the Pelnac group produced more granulation tissue and more macrophages than the Genocel group on day 14.

## 1 Introduction

Skin substitutes are widely used to treat skin defects after injury, burns, or tumor resection. Characteristics such as stability, cytocompatibility, angiogenic properties, and lack of antigenicity are required for materials to be used as skin substitutes [1,2].

Genocel (NIKKE MEDICAL Co., Ltd., Osaka, Japan) is a novel gelatin hydrogel nonwoven fabric produced by the solution-blow method using gelatin solution [3,4]. Currently, Genocel is used as a culture material to achieve homogeneous distribution and proliferation of cells. Genocel demonstrates the high mechanical strength needed to maintain a porous structure *in vitro*; cells can penetrate and homogeneously distribute into Genocel [5,6]. In addition, the fibrous and porous structure of Genocel improves the permeation of oxygen and nutrients, which increases cellular ATP production [[4], [5], [6], [7]]. When Genocel is used for cell culture, it is degraded and replaced with an extracellular matrix (ECM) as cells inside the material proliferate, and the ECM network gradually develops to maintain and enhance cell biological functions [6].

Based on these characteristics, Genocel was viewed as a promising skin substitute for wound healing. In this study, we applied Genocel to full-thickness murine skin defects and compared the healing process with that of a conventional skin substitute, Pelnac (Gunze Corp., Kyoto, Japan). Pelnac is a conventional bilayer artificial dermis with a superficial silicone layer and a lower porcine collagen sponge with a pore diameter of 60–110 μm [8]. Clinical use of Pelnac has been reported for decades [[9], [10], [11], [12], [13], [14]] and has shown good results in the treatment of full-thickness skin defects [14,15]. We compared both materials in the wound-healing process with respect to the remaining wound area, neoepithelialization, granulation tissue formation, capillary formation, and macrophage infiltration.

## 2 Materials and methods

### 2.1 Preparation of Genocel and Pelnac sheets

**Fig. 1:**
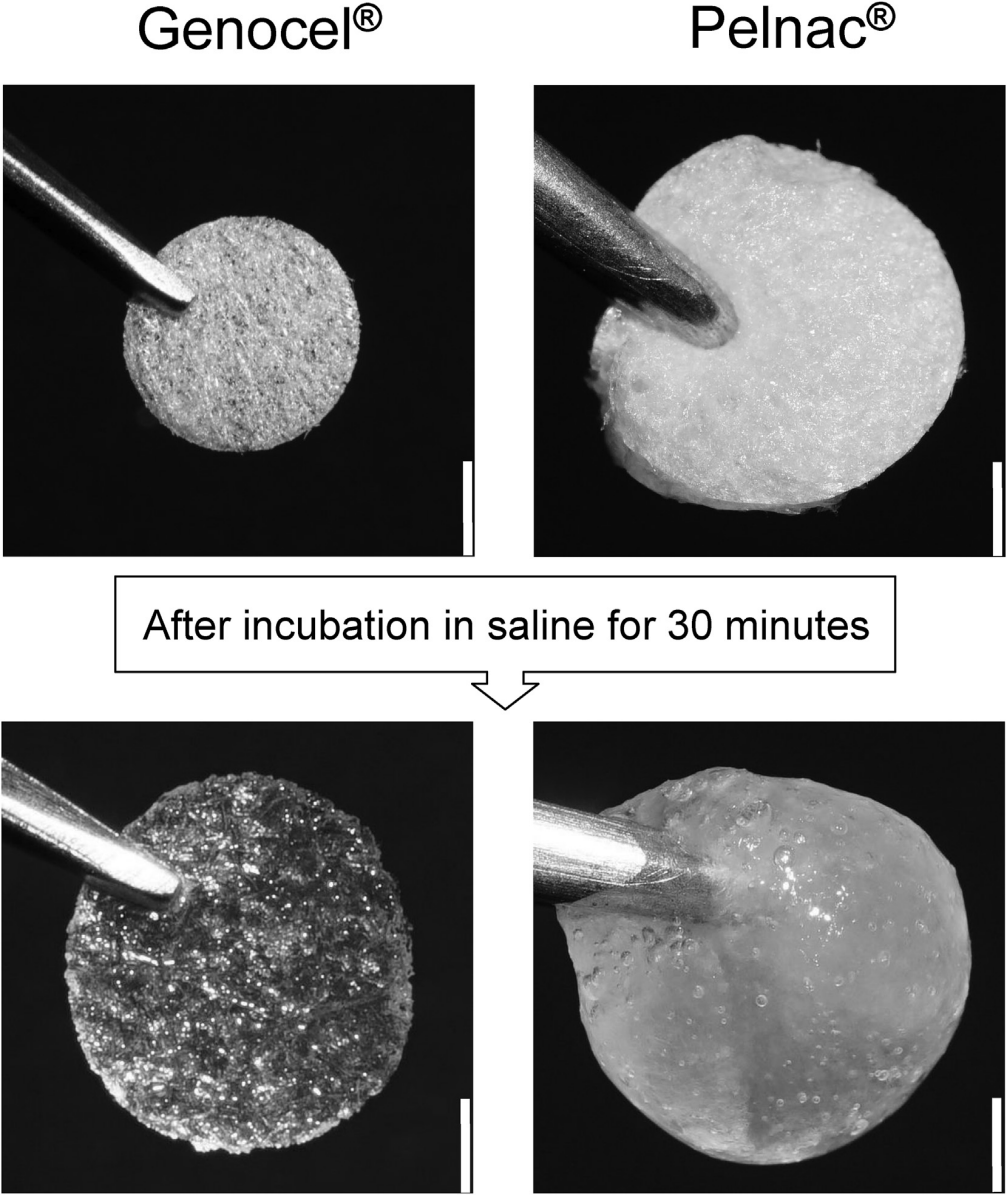
Macroscopic view of Genocel® and Pelnac® in dry condition and after a 30-min incubation in saline at room temperature. Scale bar: 2 mm.

Genocel was supplied by NIKKE MEDICAL Co., Ltd. (Osaka, Japan). Genocel sheets with 5-mm diameters were prepared and used in this study. Genocel is made of gelatin hydrogel nonwoven with porous structures [4], therefore, 5-mm pieces swelled with saline solution and reached a diameter of approximately 8 mm (Fig. 1). In this study, we used single-layer Pelnac without an outer silicon layer. Pelnac sheets 8 mm in diameter were prepared using an 8-mm biopsy punch (Kai Industries Co., Ltd., Tokyo, Japan). Genocel and Pelnac sheets were kept in saline solution (Otsuka Pharmaceutical Factory, Inc., Tokyo, Japan) for over 30 minutes at room temperature before use.

### 2.2 Preparation of animal experiments

The animal study was conducted at Kyoto University following the Guidelines for Animal Experimentation of Kyoto University, Japan. The protocol was approved by the Animal Research Committee of the Kyoto University Graduate School of Medicine (permit number: Med Kyo 20515), and the number of experimental animals used was kept to a minimum.

Thirty C57BL/6JJcl mice (male, 8–9 weeks old) (CLEA Japan, Inc., Tokyo, Japan) were fed and housed individually per cage in a temperature-controlled animal facility with a 12-h light/dark cycle and allocated to two groups: Genocel and Pelnac. One day before surgery, the hair on the back of each mouse was shaved using an electric shaver (Thrive; Daito Electric Machine Ind., Co., Ltd., Osaka, Japan) and depilated using depilation cream (Kracie, Tokyo, Japan). All painful procedures were performed under general anesthesia with isoflurane (Pfizer Inc., Kyoto, Japan) in spontaneously breathing animals. The concentration of isoflurane was kept at 1.5–2% to provide an appropriate depth of anesthesia.

In the application surgery of materials, a donut-shaped silicone skin splint (18/12 mm in outer/inner diameter, 0.5 mm in thickness; Fuji System Corp., Tokyo, Japan) was attached to the skin with binding adhesive (Aron Alpha; Daiichi Sankyo, Osaka, Japan) at first. It was then sutured and fixed to the skin using 5–0 nylon (Bear Corporation, Osaka, Japan) to prevent wound contraction. Second, a full-thickness skin defect, 8 mm in diameter, at the center of the applied skin splint was made using an 8 mm biopsy punch (Kai Industries Co., Ltd., Tokyo, Japan) and scissors. Genocel or Pelnac sheets were applied to the skin defects in each group, and then the wound was covered with a silicone mesh sheet (9 mm in diameter; SI mesh, ALCARE Co., Ltd., Tokyo, Japan) fixed to the marginal skin by suturing with 5–0 nylon, covered with gauze, and secured with surgical tape bandage (Silkytex, ALCARE Co., Ltd., Tokyo, Japan) to prevent contamination and mechanical stress. After these procedures, mice were placed in individual cages inside the institutional animal facility.

### 2.3 Evaluation of wound healing

The wound-healing process was evaluated on days 7, 14, and 21 post-surgery. Five mice in each group were sacrificed by carbon dioxide gas inhalation at each time point, and macroscopic photographs of the wounds were taken with a digital camera (Sony Corporation, Tokyo, Japan). The wound specimens, including the surrounding tissue, were harvested, fixed in 10% formalin buffer solution (FUJIFILM Wako Pure Chemical Co., Ltd., Osaka, Japan), paraffin embedded, and sectioned axially at the center of each wound according to the previous reports [16,17]. Hematoxylin and eosin (HE), Azan, and immunohistochemical staining for CD31, CD68, and CD163 antibodies was performed.

CD31, also designated as PECAM-1 (platelet endothelial cell adhesion molecule-1) is considered a reliable marker for blood vessels [18]. Thus, we analyzed the number and area of newly formed capillaries using anti-CD31-stained sections. CD68 is a pan-macrophage marker, and CD163 is a specific marker for M2 macrophages [19]; therefore, we analyzed the number of pan-macrophages and M2 macrophages using anti-CD68- and anti-CD163-stained sections, and calculated the M2 ratio using CD163+/CD68+.

For anti-CD31 staining, sections were deparaffinized and rehydrated, and heat-induced antigen retrieval was performed in ethylenediaminetetraacetic acid (EDTA) (Nichirei Biosciences Inc., Tokyo, Japan) at 98 °C for 20 min. After cooling to room temperature, sections were rinsed in distilled water and immersed in 3% hydrogen peroxide for 10 min to block endogenous peroxidase activity. Sections were rinsed in distilled water and Tris–HCl buffer (containing 0.05% Tween-20 and 0.15 M NaCl) (TBST). Sections were immersed in 3% bovine serum albumin (BSA) in PBS for 60 min at room temperature. Rabbit monoclonal antibody (ab182981, Abcam, Cambridge, UK) at a 1:10,000 dilution was applied to the sections and incubated at 4 °C overnight. The sections were then rinsed with TBST. A polymer reagent (simple stain mouse MAX PO; Nichirei Biosciences Inc., Tokyo, Japan) was applied at room temperature for 30 min. Sections were rinsed in TBST again and exposed to 3–3′-diaminobenzidine-4HCl (DAB) (Nichirei Biosciences Inc., Tokyo, Japan) and counterstained with hematoxylin.

For anti-CD68 or -CD163 staining, the staining method followed the process of anti-CD31 staining mentioned before, and rabbit polyclonal antibodies (ab125212, ab182422, Abcam) were used instead.

After staining, histological photomicrographs were obtained and analyzed using a BZ-X800 Analyzer software (Keyence Corp., Osaka, Japan).

### 2.4 Assessment of the remaining wound area

The unepithelialized wound area was manually lined on the photographs and the wound area was evaluated using the ImageJ software program (National Institutes of Health, Bethesda, MD, USA) on days 7, 14 and 21 was compared between the Genocel and Pelnac groups.

### 2.5 Assessment of neoepithelialization

Neoepithelium length was measured in HE sections on day 7, and was defined as the length of a line tracked along the epithelium from the innermost follicle to the end of the epithelium. The length was measured on both edges of the wound, and the total value was calculated.

### 2.6 Assessment of newly formed granulation tissue

The area of newly formed granulation tissue in the wound above the muscle layer was measured on Azan-stained sections on days 7, 14, and 21. The fibrous connective tissue in granulation was stained light blue with aniline blue, distinguishing it from the dermis of the wound edge, which was stained dark blue. The area of the epidermis that developed over granulation was excluded.

### 2.7 Assessment of newly formed capillaries and the capillary area

The number and total area of newly formed capillaries on days 7, 14, and 21 were measured in sections immunostained with anti-CD31 antibody. A threshold was set for the brown tint stained with DAB, and the regions with a color density higher than this threshold were counted using the BZ-X800 Analyzer software. For the capillary area, the area in which the tubular structure of the blood vessels was visible was measured, and the sum of the areas was calculated. Both of the capillary number and area were measured in the whole area of newly formed granulation tissue, as determined by Azan staining. However multiple vessel cross-sections observed on a two-dimensional section may originate from a single vessel, we simply evaluated the number and areas of capillaries observed in the sections, according to the previous studies [16,17].

### 2.8 Assessment of macrophage infiltration

The number of pan-macrophages and M2 macrophages on days 7, 14, and 21 was counted on sections immunostained with anti-CD68 or anti-CD163 antibodies, respectively. Macrophages were counted in the area of newly formed granulation tissue defined by Azan-stained sections and counted using the BZ-X800 Analyzer software, in a similar way to the vascular measurement.

### 2.9 Statistical analysis

All data are presented as mean ± standard deviation. A t-test was used to analyze the data using Microsoft Excel (Microsoft Corp., Redmond, WA, USA). Statistical significance was indicated by the probability (*P*) values < 0.05.

## 3 Results

### 3.1 Assessment of the remaining wound area

**Fig. 2:**
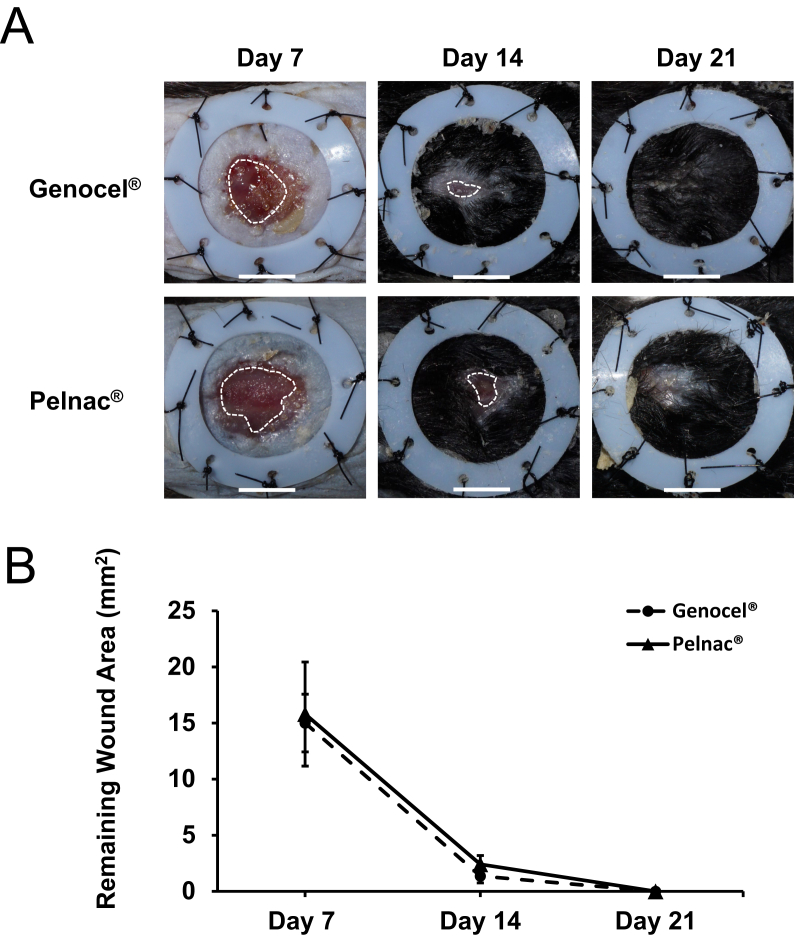
A. Macroscopic views of wounds after sheets of Genocel® or Pelnac® were applied to the skin defects created on mice, observed on days 7, 14, and 21. The white broken lines mark the edge of the neoepithelium. Scale bar: 5 mm. B. The time course of the remaining wound area on days 7, 14, and 21.

The wounds in the Genocel and Pelnac groups on days 7, 14, and 21 are shown in Fig. 2A. The time course of the wound area is shown in Fig. 2B. In both groups, the wound area steadily decreased, and the wounds were completely healed on day 21. No significant differences in area were observed between the two groups at any time point.

### 3.2 The neoepithelium length

**Fig. 3:**
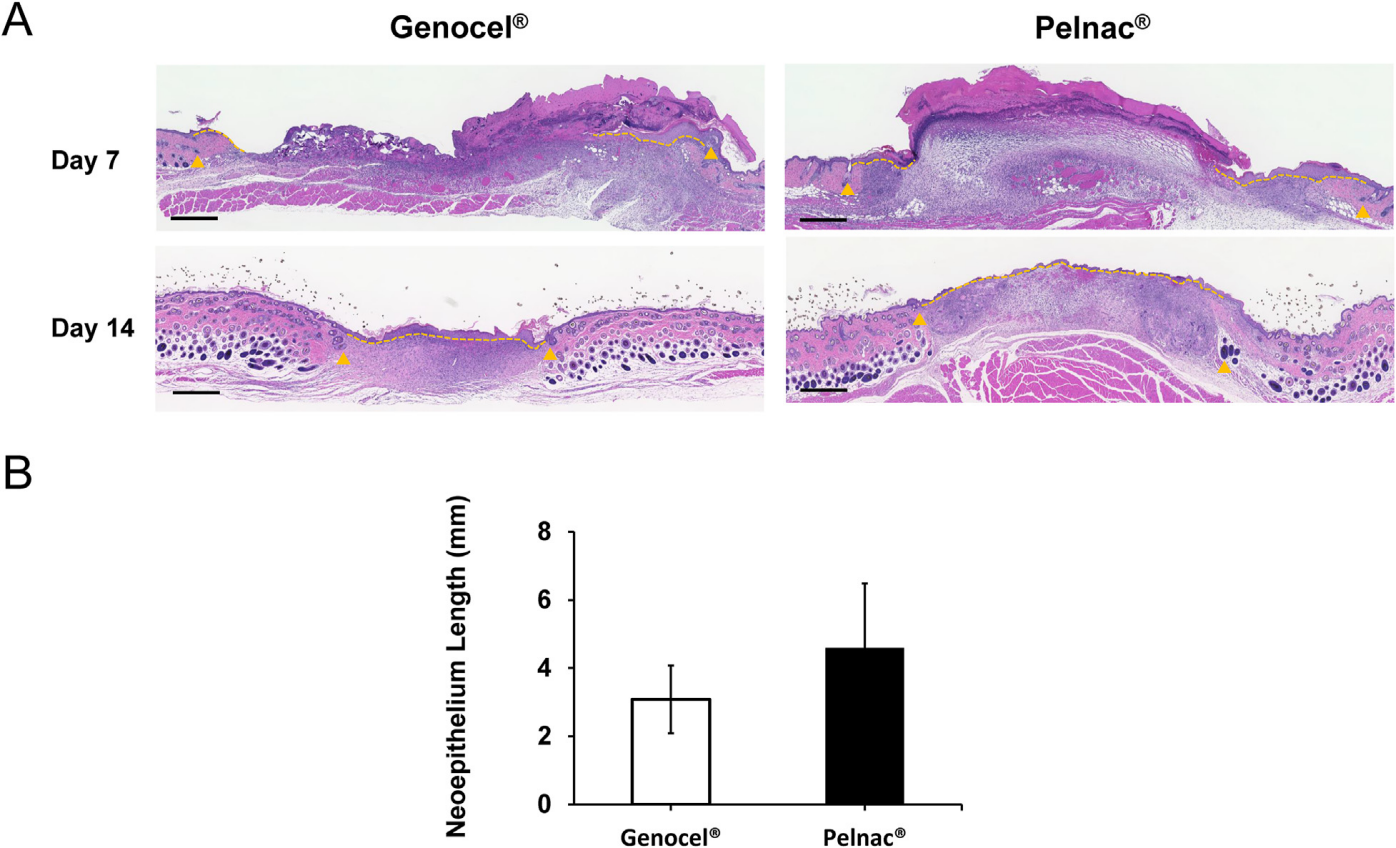
A. Micrographs of HE-stained sections on days 7 and 14. The neoepithelium length was measured between the end of epithelium and hair follicles in the wound edge. The yellow arrowheads indicate the nearest hair follicles of the original wound margin. The yellow broken lines indicate the neoepithelium. Scale bar: 500 μm. B. Comparison of the neoepithelium length on day 7. No significant difference was detected between the two groups.

Micrographs of the HE-stained sections from the Genocel and Pelnac groups on days 7 and 14 are shown in Fig. 3A, and the neoepithelium length on day 7 is shown in Fig. 3B. The remaining material was histologically observed in all cases on day 7, and epithelialization was achieved over the materials. Neoepithelium lengths were similar in the Genocel and Pelnac groups, with no significant differences observed.

### 3.3 Histological assessment of granulation tissue

**Fig. 4:**
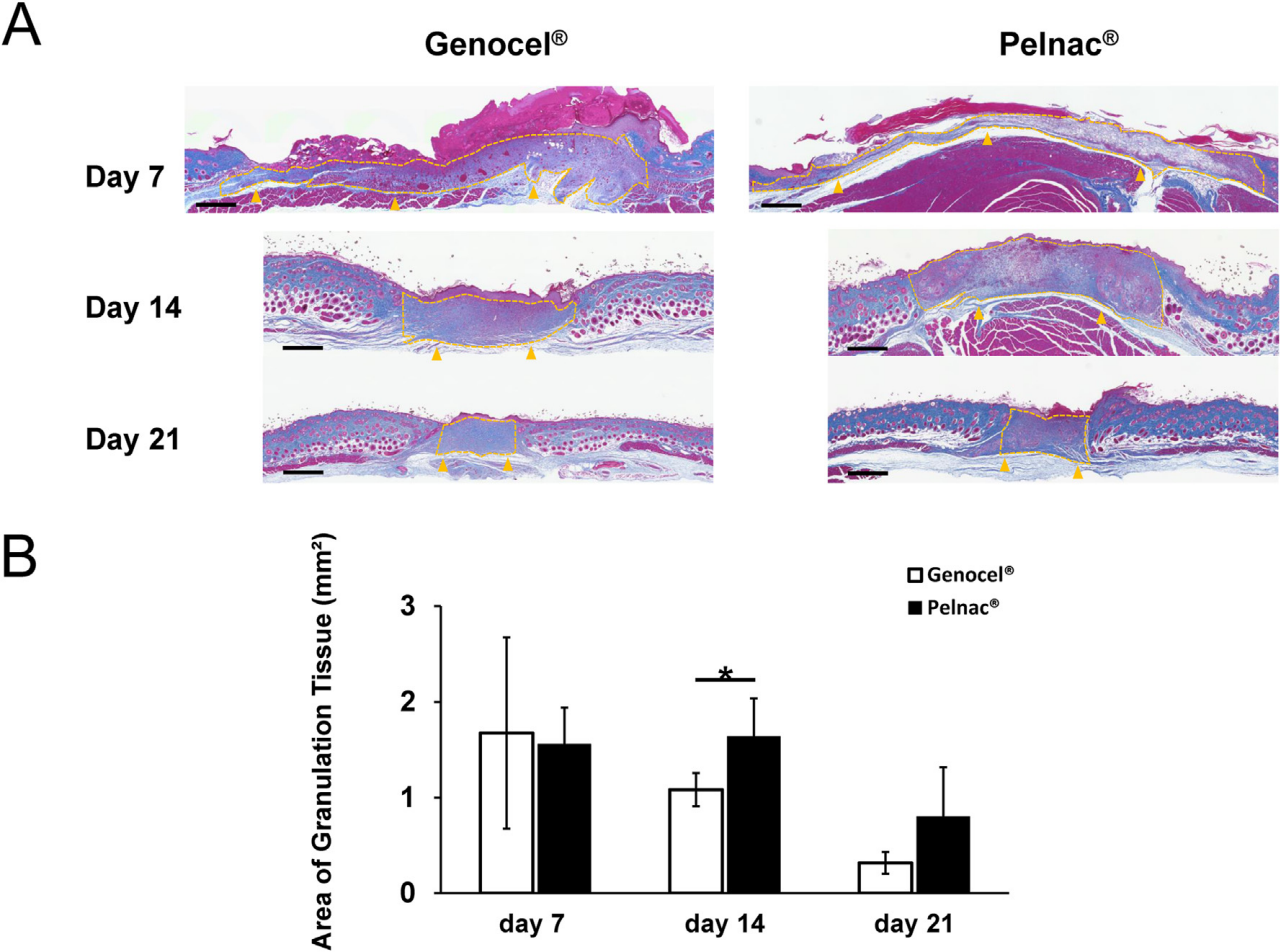
A. Micrographs of Azan-stained sections of wounds on days 7, 14, and 21. The yellow broken lines indicate the newly formed granulation tissue, and the yellow arrowheads indicate the original wound floor. Scale bar: 500 μm. B. Comparison of the area of formed granulation tissue on days 7, 14, and 21. ∗p < 0.05.

The area of the newly formed granulation tissue was evaluated in the micrographs of the Azan-stained sections (Fig. 4A). On day 14, the area of granulation tissue in the Pelnac group was significantly larger than in the Genocel group (P < 0.05). On days 7 and 21, no significant differences were observed between the groups (Fig. 4B).

### 3.4 Histological assessment of newly formed capillaries

**Fig. 5:**
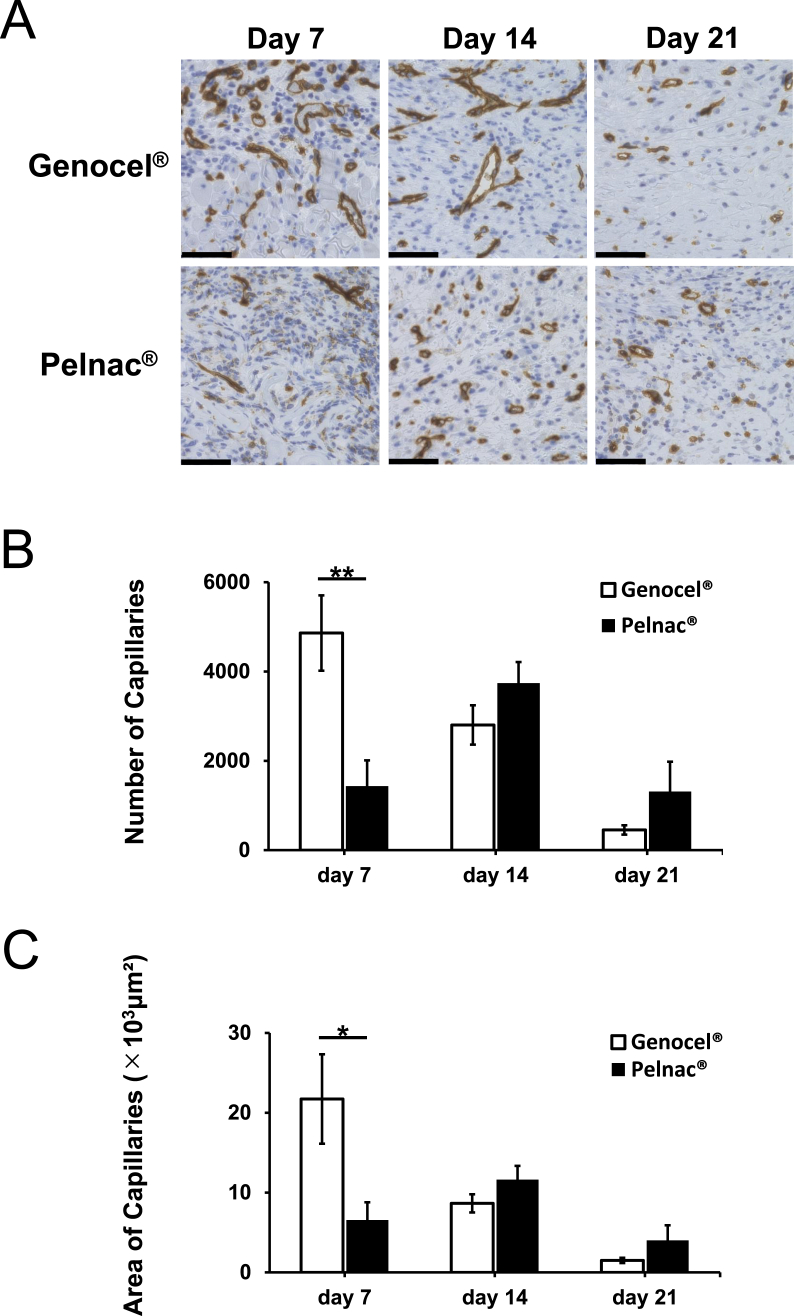
A. Micrographs of anti-CD31-stained sections of formed granulation tissue on days 7, 14, and 21. Scale bar: 50 *μ*m. B. Comparison of the number of newly formed capillaries on days 7, 14, and 21. ∗∗p < 0.01. C. Comparison of the area of newly formed capillaries on days 7, 14, and 21. ∗p < 0.05.

Micrographs of the representable areas at the middle in wound depth at wound edge immunostained with anti-CD31 antibodies are shown in Fig. 5A. The highest neovascularization in the Genocel group was observed on day 7, whereas that in the Pelnac group was observed on day 14. On day 7, both the number and area of newly formed capillaries in the Genocel group were significantly larger than those in the Pelnac group (P < 0.05). However, there were no significant differences in these variables between the groups from days 14 to 21 (Fig. 5B and C).

### 3.5 Assessment of macrophage infiltration

**Fig. 6:**
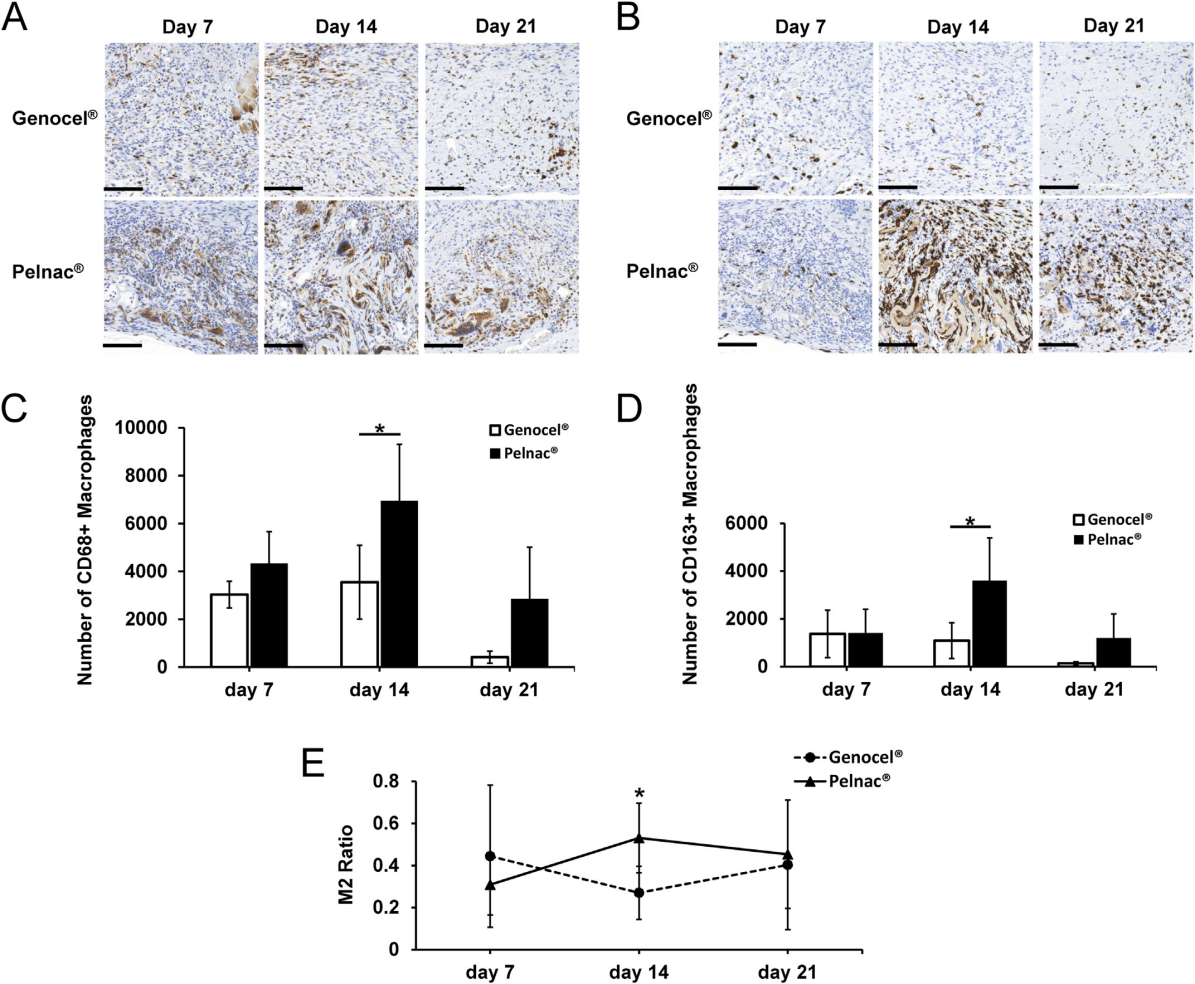
A. Micrographs of anti-CD68-stained sections on days 7, 14, and 21 from each group. Scale bar: 100 μm. B. Micrographs of anti-CD163-stained sections on days 7, 14, and 21 from each group. Scale bar: 100 μm. C. Comparison of the number of CD68+ macrophages on days 7, 14, and 21. ∗p < 0.05. D. Comparison of the number of newly produced CD163+ macrophages (M2) on days 7, 14, and 21. ∗p < 0.05. E. Comparison of the M2 ratio (CD163+/CD68+) on days 7, 14, and 21. ∗p < 0.05.

Micrographs of sections immunostained with anti-CD68 or -CD163 antibodies were evaluated (Fig. 6A and B). The number of pan-macrophages and M2 macrophages, as well as the M2 ratio in the Pelnac group, were significantly higher than in the Genocel group on day 14 (Fig. 6C, D, E) (p < 0.05).

## 4 Discussion

In this study, we explored the effect of Genocel on wound healing when applied to skin defects, and we evaluated the feasibility of using Genocel as a skin substitute. We applied the Genocel and Pelnac sheets to skin defects created in mice, and on days 7, 14, and 21, we analyzed the wound in terms of remaining wound area, length of neoepithelium, newly formed granulation tissue, capillaries, macrophage infiltration, and M2 ratio. We found that the Genocel group produced more neovascularization, with a larger vascular area, than the Pelnac group on day 7. In contrast, the Pelnac group had more granulation tissue formation, produced more M2 macrophages, and showed a higher M2 ratio than did the Genocel group on day 14.

A three-dimensional scaffold provides sufficient space and stimulates growth and development of vascular endothelial cells [[20], [21], [22], [23], [24], [25]]. Genocel is a three-dimensional gelatin scaffold that provides cells with space for proliferation, migration, and differentiation [6,26]. The high permeability of oxygen and nutrients in Genocel maintains the survival and biological functions of the cells. As the components are gradually degraded and remodeled into the ECM, mesenchymal stromal cells continually infiltrate Genocel [6]. This may explain why the Genocel group formed more capillaries with a larger area than the Pelnac group on day 7.

Macrophages play an important role in tissue repair, changing their phenotypes and thereby affecting their function. Activated macrophages are usually divided into two categories: M1 and M2. Both M1 and M2 macrophages are closely associated with the inflammatory response. M1 macrophages are capable of pro-inflammatory responses and produce pro-inflammatory factors that mainly mediate the tissue-destructive phase. In contrast, M2 macrophages take effect mainly during the anti-inflammatory response, which are responsible for the tissue-reparative phase [[27], [28], [29], [30]]. Through this plasticity, macrophages regulate inflammation and tissue remodeling, promote tissue repair, and transition to the proliferative phase of healing [30].

The large number of M2 macrophages in the Pelnac group on day 14 indicates that the transition from the inflammation phase to the proliferative and remodeling phases in tissue repair proceeded effectively. In the Genocel group, macrophage infiltration was modest, and tissue regeneration was retarded, indicating that Genocel may not be as effective as Pelnac in inducing granulation and macrophages.

The reason for the relatively inferior performance of Genocel as a skin substitute, compared to Pelnac, may be the difference in its source materials. Skin substitutes used to regenerate skin defects, the so-called artificial dermis, are usually composed of collagen, a major component of the dermis. The advantage of collagen is its high affinity for tissues and low antigenicity. The collagen sponge also spontaneously converts into large amounts of synthesized connective tissue matrix, similar to the true dermis, along with an inflammatory response when used *in vivo* [8,11]. In contrast, Genocel is composed of nonwoven fabrics of dehydrothermally cross-linked gelatin, instead of a collagen sponge. Gelatin molecules are smaller in molecular weight and degrade more rapidly than collagen [31], so they may be unable to maintain their structure long enough for granulation to occur. This may explain the advantages of Pelnac in inducing granulation and macrophages on day 14.

As Genocel has an excellent ability to induce early stage neovascularization, it may be an effective treatment option for chronic wounds such as venous leg ulcers or diabetic foot ulcers. Further studies using diabetic mice are necessary to confirm the effectiveness of Genocel. In addition, it may be necessary to optimize physical properties to improve granulation formation after Genocel implantation.

This was the first study to confirm the usefulness of Genocel as a skin substitute in murine experiments. Genocel was comparable to Pelnac in its ability to promote wound closure and epithelialization. We expect Genocel to be a new and better option for clinical wound treatment in many cases.

## 5 Conclusions

We compared Genocel and Pelnac for wound healing after implantation in a murine skin defect model. Therefore, the two materials displayed comparable behavior, and Genocel accelerated capillary formation in the early phase; however, Pelnac was superior in granulation formation and macrophage infiltration. The results indicate that Genocel can be used as a new skin substitute, especially for chronic wounds such as venous leg ulcers or diabetic foot ulcers. Future studies using diabetic mice and optimization of the physical properties of Genocel are required.

## Declaration of competing interest

All authors declare no conflict of interest in association with the present study.
